# Transactions of the Pathological Society

**Published:** 1841-02-06

**Authors:** 


### ORIGINAL COMMUNICATIONS.

### TRANSACTIONS OF THE PATHOLOGICAL SOCIETY.

*January*, 1841.

*Comparative Pathology.*

Dr. Hallowell presented the uterus of a lioness which had been ruptured during the act of parturition. The keeper had inflicted a number of hard blows upon the abdomen of the animal with a long pole, to which a rod of iron was attached. One of the cubs was found in the vagina; the other two were reduced to putrilage, and occupied the horns of the uterus. He also presented two specimens of tuberculous disease; one of these was the lungs of the *Harpyia destructor*, a species of large eagle from South America, remarkable for the great development of the lower extremities; the tarsi measured four inches in circumference; tuberculous matter was deposited upon the mucous surface of the large, as well as the smaller ramifications of the bronchial tubes, so that a probe introduced into the cavity of the former was made to pass with some difficulty into the trachea. The other specimen belonged to a species of Ateles, or long-tailed monkey from South America. The upper lobe of the right lung presented a large anfractuous cavity, lined in part by a false membrane; the lower lobe was greatly enlarged, and much heavier than the corresponding lobe of the opposite lung; it was completely infiltrated with tuberculous matter; the bronchial glands were also tuberculous.

---

*Some Account of the habits and post-mortem appearances of a Chimpanzee, (Simia Troglodytes,* Linn.,) which died in Philadelphia.* By Edw. Hallowell, M. D. *Presented, with the Specimens, to the Pathological Society of Philadelphia.*

The subject of the present notice was brought in the Saluda from Western Africa, in the summer of 1839.

She was caught about twenty miles from Monrovia, by the natives, who shot the mother, and found the young animal clinging to her breast. She remained with her captors several years, when she was sold to one of the colonists, of whom she was purchased by Dr. Goheen, who brought her to this country, after remaining six months in the colony. At this time she was supposed to be about four years

---

* *Syn. Simia troglodytes*, Blumenbach, Manuel d'Histoire Naturelle, tom. i. p. 82.

*Troglodytes chimpanzee*, *Troglodytes niger*, Geoff. St. Hilaire, Annales d'Histoire Naturelle, tom. xxx. p. 87.

*Jocko*, Buffon, Hist. Naturelle, tom. xiv., fig. 1., p. 72.

*Pongo*,† Buffon, Supplement, tom. vii. p. 2.

*Pongo*, Audebert, Hist. Nat. des Singes et des Makis, pl. 1. fig. 15.

*Simia troglodytes*, Cuvier, Regne Animale, tom. i. p. 89, 2d ed.

*Troglodytes niger*, Desmarest, Mammalogie, p. 49.

*Chimpanzee*, *Black orang*, *Pigmy; Angola Ape*, Vulgo.

† The term Pongo is now reserved exclusively for the *orangs* inhabiting the islands of Borneo and Sumatra, and is applied more particularly to the adult *orang*, *Pongo Wurmbii*, Geoff., which is considered by Cuvier, Owen, and others, as identical with the *Simia satyrus* of Linnæus.

old. A short time after her arrival she was deposited in the Philadelphia Museum, and was subsequently taken to Columbia, Pa., where she was sold for the sum of three thousand dollars. She was afterwards exhibited in New York, Philadelphia, and other northern cities. Not having seen her during life, I can say nothing of her habits from personal observation; but the following account of them, contained in a letter from my friend S. S. Haldeman, Esq., a distinguished naturalist of Columbia, may not be uninteresting.

"*Dear Sir*,—Agreeably to your request, I furnish you with a few notes on the habits of the Simia troglodytes, Lin., brought to this country by Dr. S. M. E Goheen, physician to the mission on the Gambia. Having observed the animal yourself, it is unnecessary for me to say any thing respecting the external characters; but I may mention that Dr. Traill's description applied to this animal in every respect, except that the hair upon the head was longer than in the Liverpool specimen, and the abdomen was not bare, although it had been, six months previously, as I learned from Dr. Goheen. The size of the two was nearly the same, Jenny having been between thirty and thirty-one inches high, in August, 1839.

"She was afraid of dogs, which she would beat off with a stick, or at which she would throw stones, with considerable force; but in an awkward, ill-directed manner. A large stuffed Iguana caused her to show the greatest terror, from which we may infer that they have cause to fear the serpents in their native forests.* She generally walked upon all fours, with the callous knuckles of the anterior extremities applied to the ground, except when she carried any thing, and then she seemed to prefer the upright position. She appeared to feel uncomfortable towards evening, and would place a blanket around her shoulders, in the manner of a shawl; when, if she had occasion to move, she would rise, as her hands were employed in holding her blanket. She kept awake during the day, and slept at night, being very careful to have herself well covered in her bed. She acquired from the negroes a habit of wearing a cloth around the loins, and whenever she could get any thing suitable, would apply it for a half day at a time.

"Her passion for imitation was very strong, and it may be worth mentioning that the phrenological organ was very full. This faculty gives one a strong bias to overrate the intelligence of the animal, as it is difficult to see certain actions performed, without referring them to the mental source which would give rise to them in ourselves. Thus I have seen Jenny imitate (from memory) a washerwoman, by standing beside a vessel of water with a cloth in her hands, which she would soap, dip, rub a while with both hands, wring out in the proper manner, and finally open out. She was very averse to putting her hands in cold water. She was a long time learning to unlock her chain; because, although she could insert and turn the key until it came in contact with the bolt, she appeared unconscious that a little more force should be applied at that point. She was very careful to prevent her chain from becoming entangled with sticks; and if it were purposely done, she would exercise much tact in disentangling it. When at liberty, instead of allowing her chain to drag along the ground, she carried it upon one arm, going upright, or with one hand applied to the ground. She refused scarcely any thing eatable, preferring sweet food, having no objection to meat, and relishing fried salt ham. She was fond of tea, coffee, and wine, and would smoke a pipe as if she enjoyed it,—differing in this respect from the *Papio mormon* in a London menagerie, which would not smoke without the prospect of a glass of spirits. She used a knife, fork, and spoon, and drank from a saucer or tumbler, without risk to the latter—her hand being large enough to grasp an object three inches in diameter. In drinking she did not put her snout in the glass, but placed its edge between her lips. Bright objects, such as a metal spoon, pleased her, and she would frequently scour them, but more I suspect, to imitate the action, than to brighten the metal.

"The disposition of this animal was very mild, and she was very fond of romping with negro children, her chief playmates in Africa. When at play, her countenance would sometimes assume every mark of pleasure. When tickled, she emitted a kind of laugh from the throat, unaccompanied by any voice; but if pleased, she signified her satisfaction by a succession of sounds in a low key, like that of the English letter *u* in the syllable *but;* thus, *u u u u*, at the rate of about six impulses in a second of time.

"Notwithstanding her mild temper, she would occasioally show all the vagaries of a spoiled child,—such as pouting, getting in a passion, and crying, or throwing away her food, or whatever was within her reach, and at such times refusing to be pacified with sweetmeats, or by gentle means. A free use of the whip on these occasions, was the only method to make her conduct herself properly. She

* The keeper, Mr. Elisha Hansen, who is quite an intelligent man, stated to me that she always manifested a great dread of serpents and tortoises; the small, as well as the larger species of the latter, were objects of aversion. Among the reptiles exhibited at the menagerie in New York was a living horned toad, (*Phrynosoma cornuta*, Wieg.;) when first observed by her she betrayed marked signs of fear, approaching it cautiously, and then retreating from it; she gradually, however, assumed more courage, and at length touched it with her hand: finding it harmless, she ventured still further, and taking it by the tail, examined it first on one side, and then on the other.—E. H.

sometimes killed flies by clapping her palms forcibly together as they flew past her.

"I observed Jenny, from time to time, out of doors, for a month, and when she had not purposely been taught any tricks. From these observations I formed the opinion that she possessed the intelligence of a child from sixteen to eighteen months old, as at this age children have not the faculty of language, and do not attempt to articulate;* but they are sufficiently observant to imitate actions.† I will give a single instance, from personal observation, to illustrate the intelligence of this animal. She was eating broken cherries in milk, holding a spoon in one hand, and the saucer in the other. When the food was nearly exhausted, it became difficult to fill the spoon, which she then used as a fork; but it was not very suitable, and the last morsel would not stick to it, when she tried to get it into the spoon again, but without success, the morsel sliding up the side of the saucer until it was nearly ready to fall over the edge. Of this she appeared to be aware, and was on the point of placing it in the spoon with her lips, but a word prevented it; the cherry was too precious to be lost, and it would fall over the edge rather than enter the spoon. In this predicament she raised the spoon, and with its back pushed the morsel from the edge to the centre of the saucer, apparently that she might have room to get it into the spoon. It approached the edge again, and was pushed back as before, when she was allowed to pick it up with her long flexible lips.

"The canine teeth in this subgenus are fully as large as in the carnivora, and I have reason to believe they are employed defensively. They are doubtless of service in breaking open husks, or splitting decayed wood for the purpose of getting insects. It is not improbable, judging from their habits in confinement, that they are partially carnivorous in a state of nature.

"The Linnæan name Simia has been very improperly dropped by most naturalists, when it should have been retained for the most typical subgenus of the family; and this I believe to be Troglodytes of Geoffroy. In case Pithecus is considered as the typical subgenus, the name Troglodytes must be changed, being preoccupied in ornithology; and the animal noticed above might be called Chimpanzee troglodytes, with the citation of Linnæus as the first author who characterized it, under the specific name it now bears.

* The Simiadæ would not articulate if they were provided with vocal organs, because they have not he faculty of language.

† It is evident that the desire to imitate conduces very much to the advancement of the child; but when this faculty is so highly developed in a quadruped, we are led to wonder of what use it can be in their economy. To determine this, it will be necessary to study the animals in their native state.

"I have an accurate portrait of Jenny, the size of life, which I had painted whilst she remained in Columbia. If you have any use for it, it is at your service.

"Respectfully, yours, &c.

"S. S. Haldeman.

"*Wasp Hall*, 24th July, 1840.

"P. S.—By a reference to a number of 'Africa's Luminary,' a paper published in Liberia, I find that Jenny was two feet four inches in height, about the 5th of April, 1839, and was supposed to be about four years old."

Until September, the health of "Jenny" continued perfectly good; she was indeed quite fat, and her spirits were always very lively; in the latter part of that month she was attacked with diarrhœa, which lasted four days, the discharges were slimy and bloody, but whether painful was not observed. About a week before Christmas, she was attacked for the first time with cough; she had been kept in a warm room, but the temperature does not appear to have been regulated; the cough increased in frequency, and was in a short time accompanied with a profuse expectoration. Emaciation now advanced rapidly, her breathing became much oppressed, and there was a total loss of appetite. She never had hemoptysis. A week before her death she was attacked with vomiting, which lasted a day only. During the last days of her illness her sufferings were extreme; her keeper, who appears to have been much attached to the animal, states that her countenance exhibited marks of the greatest distress, and that she frequently extended her hands towards him as if imploring relief. She died on Tuesday, the 27th of April, about four months from the invasion of the cough. During this time she had no diarrhœa. The following are the notes of the autopsy: (When I first saw the animal, the skin had been removed, and the extremities were separated from the body. The thorax had also been opened by several medical gentlemen, but the parts were little disturbed.)

*Brain.*—But little blood upon the surface of the dura mater; veins of pia mater moderately distended; arachnoid moist, transparent, no effusion beneath; the pia mater is much injected both upon the base and upon the convex surface of the brain; on the posterior part of the left hemisphere, there is a slight ecchymosis; no tuberculous granulations; convolutions more flattened than in the human subject; cortical substance of a deep ash colour, not injected, about a line and a quarter in depth; medullary substance pale and quite firm; no serosity in cavity of ventricles; thalami nervor. optic. corpora striata, pons and medulla oblongata apparently of normal consistence.

*Neck.*—Uvula, pharynx and tonsils much injected; in the right tonsil is a small grayish ulcer about a line in diameter; lining membrane of larynx pale, not ulcerated.

*Thorax—left lung.*—Pleura free; no tubercles; the lung itself is of a reddish brown colour externally; the upper lobe is engorged and contains a few miliary tubercles in its summit; the lower lobe is covered with a delicate false membrane, of a light yellow colour, easily separated from the surface of the lung; the pleura beneath is much injected; the tissue of this lobe is more condensed than that of the upper, and is of a paler colour; in its centre is a small abscess about the size of a pea, containing softened tuberculous matter. The pleura contains about a gill and a half of pus of a dirty white colour, and of cream like consistence; its surface is covered with false membranes, which adhere to it but slightly. In the upper part of the *right* lung is a large tuberculous cavity; it occupies a considerable portion of the upper, and nearly the whole of the middle lobe; no lining membrane is observed in it, its walls being formed by the tissue of the lung itself; the lower lobe is friable, and of a grayish white colour throughout, the tuberculous deposit existing here in a state of infiltration. The bronchial glands are much enlarged and tuberculous; one of them measures an inch and a quarter in length by three-fourths of an inch in breadth; another just within the bifurcation of the trachea, is an inch and a half in length; it is more or less softened; lining membrane of trachea and bronchi much injected, but not thickened. Several small ulcers are observed near the origin of the latter.

*Heart*, two and a quarter inches in length, measured upon its anterior face from origin of aorta; circumference at base three inches; there is a considerable quantity of fat about the origin of the large vessels, and at base of auricles, also at its apex; valves healthy; pericardium pale, not containing any fluid.

*Abdomen.*—Peritoneum pale; intestines pale and moderately distended; a considerable quantity of fat between folds of omentum; no fluid in abdominal cavity. *Liver*, six inches by four, of a slate colour above, except in a narrow space along its anterior margin, where it is brownish red, marked with yellow; internally it is of a pale flesh colour, and of good consistence, not fatty; at its left extremity, near the edge, upon the upper surface of the liver is a tuberculous deposit six lines in length by four in breadth; it penetrates about four lines into the tissue of the gland; gall bladder distended with bile of a bright orange colour; its coats appear much thicker than in the human subject. *Spleen*, four and a half by two inches, of a light slate colour externally; tissue reddish brown and friable, containing numerous tubercles, many of which are in a softened state. *Pancreas* healthy. The *stomach* contains a moderate quantity of fluid, without perceptible odour; mucous membrane pale, and apparently of good consistence for the most part; strips one inch and a quarter being obtained along the lesser curvature, and one inch in the greater; in the great cul-de-sac, in a space about two inches in extent, it is much softened; the softening appears to extend to the cellular and muscular coats, both of which are reduced into a pulp by scraping with the finger nail; the softened portion is of a grayish white colour, with several blackish spots near its centre; the mucous membrane surrounding it presents a slight rose coloured tint, with a few small arborizations. The *small intestine* contains some yellowish matter of the consistence of thin gruel; the mucous membrane is perfectly pale throughout; strips four lines in length are obtained in the duodenum and five or six in the ileum; the mucous cryptæ of the duodenum in a space five inches in extent from pylorus are quite apparent; they are about a fourth of a line in diameter, each having a minute central orifice, hardly perceptible to the naked eye; the isolated follicles in other parts of the intestine are scarcely visible; the mucous membrane in an extent of six feet from pylorus is perfectly free from ulceration, as is also the ileum for twenty and a half inches from cœcum; in the intermediate space eleven ulcers are observed, of a rounded form for the most part, with thickened edges; the smallest is about the size of a pin's head, the largest measures eight lines in length by four in breadth; several of the ulcers are quite superficial, not penetrating beyond the mucous coat; they are situated in the upper part of the space indicated; the others, or those nearer the cœcum, are deeper, the submucous tissue being more or less thickened, and in places destroyed, leaving the muscular coat exposed; in several the ulceration has penetrated as far as the peritoneal coat, very slight traction being sufficient to cause its rupture; the *plaques of Peyer*, of which thirty were counted, are generally healthy; five of them present ulcerations of various sizes, and one appears to have been entirely destroyed; the surrounding mucous membrane as well as the ulcers themselves is perfectly pale; but three granulations are observed throughout the entire tract of the intestine; one of these is situated at the inferior extremity of one of the plaques of Peyer, presenting a slight ulceration upon its surface; the other two are larger, and are about two feet and a half from the lower extremity of the ileum; they are of a whitish yellow colour, and are seen distinctly through the peritoneal coat; the mucous membrane above them is quite pale, and apparently healthy, as well as the surrounding membrane; they are about a line apart, and one of them (the posterior) is within three lines of the attachment of the intestine to the mesentery. The *large intestine* contains a considerable quantity of yellowish fœcal matter; the mucous membrane is a deep slate colour, which is perhaps the natural appearance; it is apparently of good consistence, yielding strips about eight lines in length; the mucous follicles are greatly developed, being at least three-fourths of a line in diameter; no lumbrici are observed in any part of the intestines. The *mesenteric glands* are much enlarged, several of them are of the size of an almond; they contain a quantity of white caseous looking matter, more or less softened. *Kidneys*,—two and a half inches in length by two in breadth; colour pale red externally; the cortical substance contains several tubercles, the largest about a line in diameter. Bladder contracted; mucous membrane pale. *Uterus* and its appendages healthy.

The anatomy of the animal, so far as this was subsequently examined by my friend Dr. Grant, a zealous and skilful anatomist of this city, and by myself, corresponded in most respects with the account given by Dr. Traill, in the third volume of the Memoirs of the Wernerian Society. We noticed, however, several differences. There were fourteen pairs of ribs,* whereas in the specimen described by Dr. Traill and other anatomists, there were but thirteen. The "very small heart shaped lobule loosely attached to the posterior part of the great fissure on the concave side of the liver," was not observed in our subject; the form of the uterus resembled more the human; although much less distinctly pyriform, it was evidently broader at the fundus than at the apex; the palmaris longus was very distinct in one of the arms of our animal, as in that of Dr. Traill; there was no flexor longus pollicis, but a small tendinous slip detached from the flexor profundus supplied its place. There was no plantaris, but the popliteus was larger in proportion than in the human subject. The extensor longus digitorum sent tendons to four of the fingers, but none to the little finger, corresponding in this respect with the animal dissected by Tyson. There was but one extensor longus pollicis. In contact with the tibialis anticus was a muscle which does not exist in the human subject; it is inserted into the base of the metatarsal bone of the thumb; this muscle, according to Mr. Owen, exists also in the orang utan, and is found in the other simiæ; the soleus had but one origin arising from the head of the fibula; the beautiful but somewhat complicated mechanism for the flexion of the toes was precisely such as is described in the paper above mentioned. Very careful admeasurements were made of the different parts of the skeleton, but making allowance for the greater size of the animal, they did not differ materially from those published by Mr. Owen, in his admirable paper on the osteology of the chimpanzee and orang utan.† The external appearance as stated by Mr. Haldeman, differed in no respect from that observed by Dr. Traill, except that the abdomen was sparsely covered with hair, and that the hair on the back of the head was longer, measuring two inches in length.

The height of the animal was thirty-three inches.

*Observations.*—Comparative Pathology has elicited but little attention in this country, nor even in Europe has it been much investigated, except in relation to the diseases of the domestic animals. Many facts however are recorded in the compendium of Otto, and a number of highly interesting autopsies may be found in the proceedings of the Zoological Society of London; among the latter is one of a *Simia Satyrus*, and also of a male Chimpanzee, by Mr. Owen. Mr. Reynaud, a distinguished physician of Paris, has investigated with much care the subject of Phthisis,* as it occurs in the quadrumanous animals, nearly all of which, in *confinement*, die of that disease. In the greater part of the cases observed by him, the tuberculous deposit was not confined to the lungs, but existed in other organs, and in several in a degree of intensity but seldom observed in the human subject. The organs most frequently affected were the bronchial glands, the spleen, the liver,† and the kidneys, the latter nine times out of ten; tubercles were found in the spleen eight times in the same number. Many were in a softened state, while no such appearance was observed in the lungs in several cases; hence, it was inferred that in these cases they existed in the spleen prior to their development in the pulmonary tissue. Tuberculous infiltration of the lung would appear to be not uncommon in these animals, M. Reynaud having met with it several times. It will be remembered that it existed in both the specimens presented to the Society. It is remarkable that in none of the cases observed by M. Reynaud, were ulcerations found either in the intestines or in the trachea or bronchial tubes. The symptoms in the chimpanzee whose history I have reported, do not appear to have been as numerous as would have been observed in a case of equal intensity in the human subject; no mention is made of night sweats, and there was no hemoptysis, nor was there any diarrhœa, except in the commencement, notwithstanding the presence of

* Two of the ribs were fractured, (the seventh and eighth, left,) and had united in the usual manner.

† Transactions of the Zoological Society of London, vol. I. part IV.

* Archives Generales de Medecine, tom. XXV.

† In six autopsies which we have made within a twelvemonth, of children who have died of tuberculous meningitis, tubercles were found in the liver five times; (three times in the sub-peritoneal tissue, and twice in its substance.) The granulations beneath the peritoneum could hardly escape the notice of one familiar with their appearance beneath the arachnoid, but unless the liver be cut in repeated slices, the tuberculous deposits in its tissue being for the most part small, and few in number, might readily pass unobserved. In one hundred and twenty autopsies of adults who died of phthisis, M. Louis found tubercles in the liver but twice.

numerous ulcerations in the small intestine. It should not be forgotten, however, that the only testimony on this point, is that of the keeper, and should therefore be received with a certain degree of reserve. It should also be stated, that in all the animals observed by M. Reynaud, the cough was dry, expectoration not having been noticed in a single instance; this fact, taken in connexion with the absence of ulcers in the trachea and bronchial tubes, would seem to confirm the opinion of M. Louis, that their presence is due to the passage of the secretions over the lining membrane. In conclusion, we cannot but hope with Mr. Hodgkin, that increased attention may be given to the study of Comparative Pathology, believing, as we do, that "the derangements of structures and organs in inferior animals, are calculated to throw important light on those of man."

